# A Comparative Study between *Solenopsis invicta* and *Solenopsis richteri* on Tolerance to Heat and Desiccation Stresses

**DOI:** 10.1371/journal.pone.0096842

**Published:** 2014-06-10

**Authors:** Jian Chen, Tahir Rashid, Guolei Feng

**Affiliations:** 1 Biological Control of Pests Research unit, Mid South Area, Agriculture Research Service, United States Department of Agriculture, Stoneville, Mississippi, United States of America; 2 Alcorn State University, Extension/Research Demonstration Farm & Technology Transfer Center, Mound Bayou, Mississippi, United States of America; University of Sussex, United Kingdom

## Abstract

*Solenopsis invicta and Solenopsis richteri* are two very closely related invasive ant species; however, *S. invicta* is a much more successful invader. Physiological tolerance to abiotic stress has been hypothesized to be important to the success of an invasive species. In this study, we tested the hypothesis that *S. invicta* is more tolerant to heat and desiccation stress than *S. richteri*. The data strongly support our hypothesis. *S. invicta* was found to be significantly less vulnerable than *S. richteri* to both heat and desiccation stress. Despite *S. richteri* having significantly higher body water content, *S. invicta* was less sensitive to desiccation stress due to its significantly lower water loss rate (higher desiccation resistance). After the cuticular lipid was removed, *S. invicta* still had a significantly lower water loss rate than *S. richteri*, indicating that cuticular lipids were not the only factors accounting for difference in the desiccation resistance between these two species. Since multiple biological and/or ecological traits can contribute to the invasion success of a particular species, whether the observed difference in tolerance to heat and desiccation stresses is indeed associated with the variation in invasion success between these two species can only be confirmed by further extensive comparative study.

## Introduction

The red imported fire ant, *Solenopsis invicta*, is one of the most successful invasive ants in the world, and is included in the 100 of the World’s Worst Invasive Alien Species [1].

Native to South America, *S. invicta* has been introduced into many countries and regions, including the United States, Australia, China, Philippines, Thailand, Taiwan, Hong Kong, Macau and etc., [2] and has a great potential to spread further [3]. The black imported fire ant, *Solenopsis richteri*, a closely related species to *S. invicta* [4], is also an invasive ant but with much less success. *S. richteri* has established only in the southern United States outside its native range. Both ants invaded the United States through the same entrance: the port of Mobile, Alabama [5]. *S. richteri* arrived in about 1918 and *S. invicta* in 1930s [5]. Although *S. richteri* was introduced and established more than one decade earlier than *S. invicta*, the latter has gradually displaced *S. richteri* throughout most of its distribution. Currently, *S. invicta* is found in 13 states and Puerto Rico [6]. In contrast, *S. richteri* is confined to a relatively small area along the northern boundary of *S. invicta*’s distribution range, including northern Alabama, north Mississippi and southern Tennessee [5], [7], [8].

Both *S. invicta* and *S. richteri* belong to *saevissima* species complex [9]. In the United States, they had been considered as two color variants of the same species before Buren confirmed their species status in 1972 [10]. Although complete reproductive isolation was found in their native areas of sympatry [11], hybridization does occur extensively in the United States [12]. Some differences between *S. invicta* and *S. richteri*, though maybe in some less noticeable details, may play an important role in the variation in invasion success between these two species.

Release from natural enemies has been frequently used in explaining the success of *S. invicta* in the introduced areas [13], [14], [15], [16]. However, the enemy release hypothesis alone can’t explain the success of *S. invicta* over *S. richteri*, since the latter was also released from its natural enemies after it invaded the United States. Physiological tolerance to abiotic stress has been hypothesized to be important to the success of an invasive species. Unfortunately, although heat tolerance, and water balance of *S. invicta* have been investigated [17], [18], the only comparative study between *S. invicta* and *S. richteri* on response to abiotic stresses was on cold tolerance [19]. It was found that *S. richteri* workers were more tolerant to cold than *S. invicta* [19], which may be useful in explaining why *S. richteri* is able to survive along the northern boundary of *S. invicta* in the United States, but not helpful in explaining the success of *S. invicta*. In this study we tested the hypothesis that *S. invicta* has significantly higher tolerance to heat and desiccation stresses than *S. richteri*. In addition to the survivorship of ants under heat and desiccation stresses, a detailed analysis of total water content, critical water content (the amount of water of moribund ants under desiccation stress) and water loss rates was conducted. The effect of total cuticular lipid on water loss was also investigated.

## Materials and Methods

### Ants

*S. invicta* were collected from Washington County, Mississippi and *S. richteri* from DeSoto County, Mississippi. Permission for collecting ant colonies on highway right-of-way was issued by Mississippi Department of Transportation. Colonies were maintained in an insect rearing room at 26°C. All colonies used in this study were ensured to be free of *Kneallhazia solenopsae*, a microsporidian pathogen. The social form of *S. invicta* colonies was determined using PCR on *Gp*-9 alleles. The method described by Valles and Porter [20] was used to amplify *Gp*-9 alleles. All ants used in this study were from monogyne colonies.

### Mortality Under Heat Stress

Mortality under heat stress was measured for small workers [body weight: 0.85±0.30 mg (mean ± SD) for *S. richteri* and 0.65±0.31 mg for *S. invicta*], large workers (3.87±0.57 mg for *S. richteri* and 3.99±0.65 mg for *S. invicta)* and female alates (11.25±0.87 mg for *S. richteri* and 11.33±1.16 mg for *S. invicta*). A replicate consisted of 20 large workers, 20 small workers, or 10 female alates in a 30-ml capped plastic cup. Ants were subjected to heat stress in a growth chamber. A wet cotton ball was placed in the cup to ensure that ants succumbed to heat rather than dehydration. A data logger (Onset Computer Corporation, Inc. Pocasset, MA) was placed in the growth chamber to record the temperature and humidity. After 24 h, dead ants were counted. Ants often encounter 40°C soil surface temperature [21], so three temperatures were tested (growth chamber was set to 39°C, 40°C and 41°C; however, the readings of a data logger were 37.88°C. 38.77°C and 39.22°C respectively. The readings of the logger were used for summarizing the data). Workers and female alates were tested in two separate bioassays. For each species, 6 colonies were used for workers and 8 colonies for female alates. There were 3 replicates (cups) from each colony. Pooled cross-colony data were used for analysis, there was therefore a total of 18 replicates for each category of workers and 24 replicates for each species of female alates. By using the same method, mortalities at 25°C (under no thermal stress) were also measured for ants from the same colonies.

### Mortality Under Desiccation Stress

Time to 50% mortality (LT_50_) was estimated for large and small workers and female alates. Ants were subjected to desiccation stress in a 7.5-L glass desiccator at room temperature (∼23°C). Twenty ants were placed in a plastic cup with the inner wall coated with Fluon (BioQuip Products, Rancho Dominguez, CA) to prevent escape of ants. The cups were placed in desiccators using drierite as a desiccant (W. A. Hammond Company, Xenia, OH). A data logger was placed in the desiccator to record the temperature and humidity. For small workers, mortality was checked every 2 h until there were enough data points for estimating LT_50_ values. For large workers, mortality was checked every 6 h. The mortality of female alates was recorded after they were subjected to desiccation for 24.0, 44.5, 63.0 and 68.0 h. Three colonies were used for workers and 4 for female alates of each species. There were 3 replicates for each body size of workers and 4 replicates for each colony of female alates. LT_50_ value of workers was calculated for each colony. No LT_50_ value was estimated for female alates. The pooled cross colony data were used for comparing two species of female alates, therefore there was a total of 12 replicates for each species.

### Water Loss Rate

Since CO_2_ emission will have negligible effect on mass loss rate in ants, the mass loss rate was used directly as water loss rate [22], [23]. Ants were subjected to desiccation (23.5% RH) in a desiccator at room temperature (∼23°C). Water loss rate was calculated using the following equation: Water loss rate = total mass loss/ant weight/exposure time. Ants were placed in a 100-ml cup. The inner wall of the cup was coated with Fluon to prevent ants from escaping. The ants were weighed before and after they were subjected to the desiccation stress in a desiccator. In each replicate, an average 1.90 g of workers with various body sizes or an average 0.11 g of female alates (10 ants) was used. For workers, there were 9 replicates for each species and ants from different colonies were used for each replicate. For female alates, 4 colonies were used and there were 4 replicates from each colony. The exposure time was 30 min for workers and 1345 min for female alates. The pooled cross colony data were used for analyzing female alate data. Therefore there was a total of 16 replicates for female alates.

### Total Water Content

Water content was measured for workers and female alates. Weight of 30 large workers, 30 small workers, and individual female alate were measured immediately after being separated from the colony and after being freeze-dried for 48 h at −19°C. Ants were frozen at −80°C for 1 h before being freeze-dried. Five colonies from each species were used for workers, and 8 *S. invicta* colonies and 11 *S. richteri* colonies for female alates. For workers, there were 3 replicates for each colony. For female alates, there were 7 to 30 replicates from each colony for *S. richteri* and 4 to 30 for *S. invicta*. The pooled cross colony data were used for analysis. There was a total of 15 replicates for each category of workers, 100 replicates for each species of female alates.

### Critical Water Content

The water contents of moribund ants under desiccation stress were measured. About 100 ants were placed in a 100-ml plastic cup. The inner wall of the cup was coated with Fluon to prevent ant escape. Ants were subjected to desiccation stress in a desiccator (23.5% RH) at room temperature. When an ant appeared dying (unable to right itself), it was removed from the cup and weighed immediately. The ant was then placed in a 2 ml vial, frozen at −80°C for 1 h, freeze-dried at −19°C for 48 h, and weighed. Seven *S. invicta* colonies were used for both large and small workers and 4 for female alates. Five *S. richteri* colonies were used for small workers, 4 for large workers and 7 for female alates. There was a total of 33 observations for *S. invicta* small workers, 44 for *S. richteri* small workers, 44 for large *S. invicta* workers, 45 for large *S. richteri* workers, 78 for *S. invicta* female alates and 92 for *S. richteri* female alates. The pooled cross colony data were used for comparison between species.

### Effect of Cuticular Lipid on Water Loss

For evaluating the effect of cuticular lipids on water loss rate, worker ants were first frozen at −80°C for 20 min. One gram of workers were then placed in a funnel which was blocked using glass wool. Lipids were removed by washing ants using 5 ml hexane 5 times. After residual hexane was evaporated, ants were subjected to 23.5% RH and room temperature (23°C) in a desiccator for 60 min and then weighed. There were three replicates and ants from different colonies were used for each replicate.

### Statistical Analysis

Since data on ant mortality under heat stress did not follow normal distribution, Kruskal-Wallis test was used for each temperature among 4 categories of worker ants, including large red imported fire ant (LR), small red imported fire ant (SR); large black imported fire ant (LB), small black imported fire ant (SB). Mann–Whitney U test was used for pairwise comparison between categories of workers and between species of female alates. Data on mortality of female alates under desiccation stress and critical water content of female alates were also analyzed using Mann–Whitney U test. Polo Plus (Version 2.0, LeOra Software, Petaluma, California, USA) was used to estimate LT_50_ value with a 95% confidence interval (CIs). The relative LT_50_ ratio with their upper and lower 95% confidence limits was used to evaluate the significance of difference between LT_50_ values. The significance was set at *P* = 0.05 probability level. If the 95% confidence interval of the ratio between two LT_50_ values included 1, they were not considered significantly different. The data on water loss rate, total water content, critical water content of workers and effect of cuticular lipid on water loss were normally distributed, so a *t*-test was used for comparison between two species.

## Results

### Mortality Under Heat Stress

**Figure 1:**
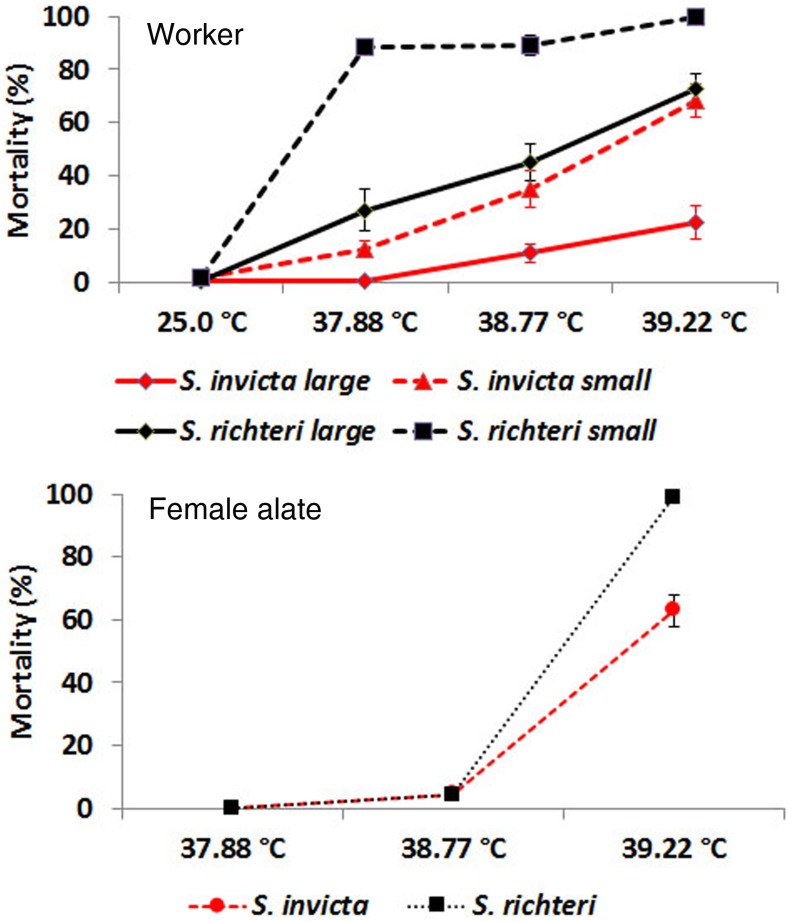
Mortalities of *S. invicta and S. richteri* workers and female alates 24 h after being subjected to heat stress.

At 25°C, there was no significant difference in mortality among large red imported fire ant (LR), small red imported fire ants (SR); large black imported fire ant (LB), and small black imported fire ant (SB). However, at 37.88°C. 38.77°C and 39.22°C, there was always a significant difference among categories of worker ants (Fig. 1, Table 1). Large workers always had lower mortality than small workers for both species (Fig. 1). At the same body size, the black imported fire ants always had significantly higher mortality than the red imported fire ants at all three temperatures (Fig. 1, Table 2). Large workers always had lower mortality than small workers for both species (Fig. 1). No mortality of female alates occurred for both species at 25°C and 37.88°C. At 38.77°C, mortality did occur for both species; however, there was no significant difference between two species (*z* = 0.53; *P* = 0.30). At 39.22°C, mortality of *S. richteri* female alates was significantly higher than that of *S. invicta* female alates (*z* = 5.47; *P*<0.0001) (Fig. 1).

**Table 1:** Results of Kruskal-Wallis test on mortalities of 4 worker categories under four different temperatures**.**

Temperature (°C)	*H*	*df*	*P*
25	4.28	3	0.23
37.88	49.53	3	<0.0001
38.77	42.09	3	<0.0002
39.22	44.35	3	<0.0003

Categories of ants include large red imported fire ant, small red imported fire ant, large black imported fire ant, and small black imported fire ant.

**Table 2:** Results of Mann–Whitney U test for pairwise comparison in mortality between worker categories under different temperatures.

Temperature (°C)	Pairwise comparison	*Z*	*P*
37.88	LB vs LR	3.84	<0.0001
	SB vs SR	5.144	<0.0001
38.77	LB vs LR	3.71	0.0001
	SB vs SR	5.54	<0.0001
39.22	LB vs LR	4.22	<0.0001
	SB vs SR	3.94	<0.0001

LR: large red imported fire ant, SR: small red imported fire ant, LB: large black imported fire ant, SB: small black imported fire ant.

### Mortality Under Desiccation Stress

**Figure 2:**
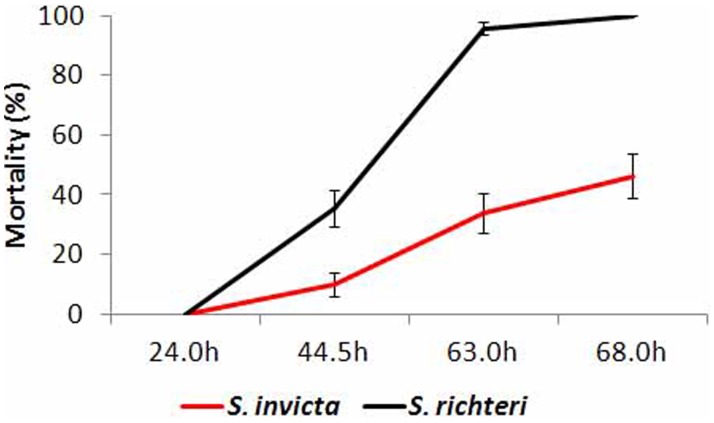
Mortalities of *S. invicta and S. richteri* female alates under experimental desiccation stress (23.5% RH at 23°C).

For both workers and female alates, *S. invicta* survived significantly longer than *S. richteri* under experimental desiccation stress. For *S. invicta*, LT_50_ values ranged from 13.65 h to 15.93 h for small workers, and 42.82 h to 47.40 h for large workers (Table 3). For *S. richteri*, LT_50_ values ranged from 10.80 h to 11.55 h for small workers and 29.08 h to 34.18 h for large workers (Table 3). For female alates, no mortality was observed at 24 h for both species. *S. invicta* female alates had significantly lower mortality than *S. richteri* after being exposed to desiccation stress for 44.5 h (*z* = 3.18; *P* = 0.0015), 63.0 h (*z* = 4.76; *P*<0.0001), and 68.0 h (*z* = 4.88; *P*<0.0001) (Fig. 2).

**Table 3:** LT_50_ values of *S. invicta and S. richteri* workers under experimental desiccation stress (23.5% RH at 23°C).

Species	Colony	Body size	Slope (± SE)	LT50	95% CI
*S. invicta*	A	small	16.21 (1.55)	15.93	15.51–16.34
		large	11.31 (1.00)	42.82	41.35–44.00
	B	small	13.28 (1.25)	13.68	13.25–14.11
		large	11.05 (0.68)	47.4	46.48–48.37
	C	small	16.20 (1.54)	13.65	13.27–14.01
		large	8.00 (0.53)	44.29	43.18–45.42
*S. richteri*	D	small	13.62 (1.28)	11.24	10.86–11.61
		large	8.58 (0.50)	29.08	28.14–30.06
	E	small	12.99 (1.23)	11.55	11.15–11.94
		large	7.58 (0.48)	33.99	32.81–35.27
	F	small	10.35 (1.00)	10.8	10.36–11.36
		large	9.53 (0.52)	34.18	33.38–34.99

### Water Loss Rate

**Figure 3:**
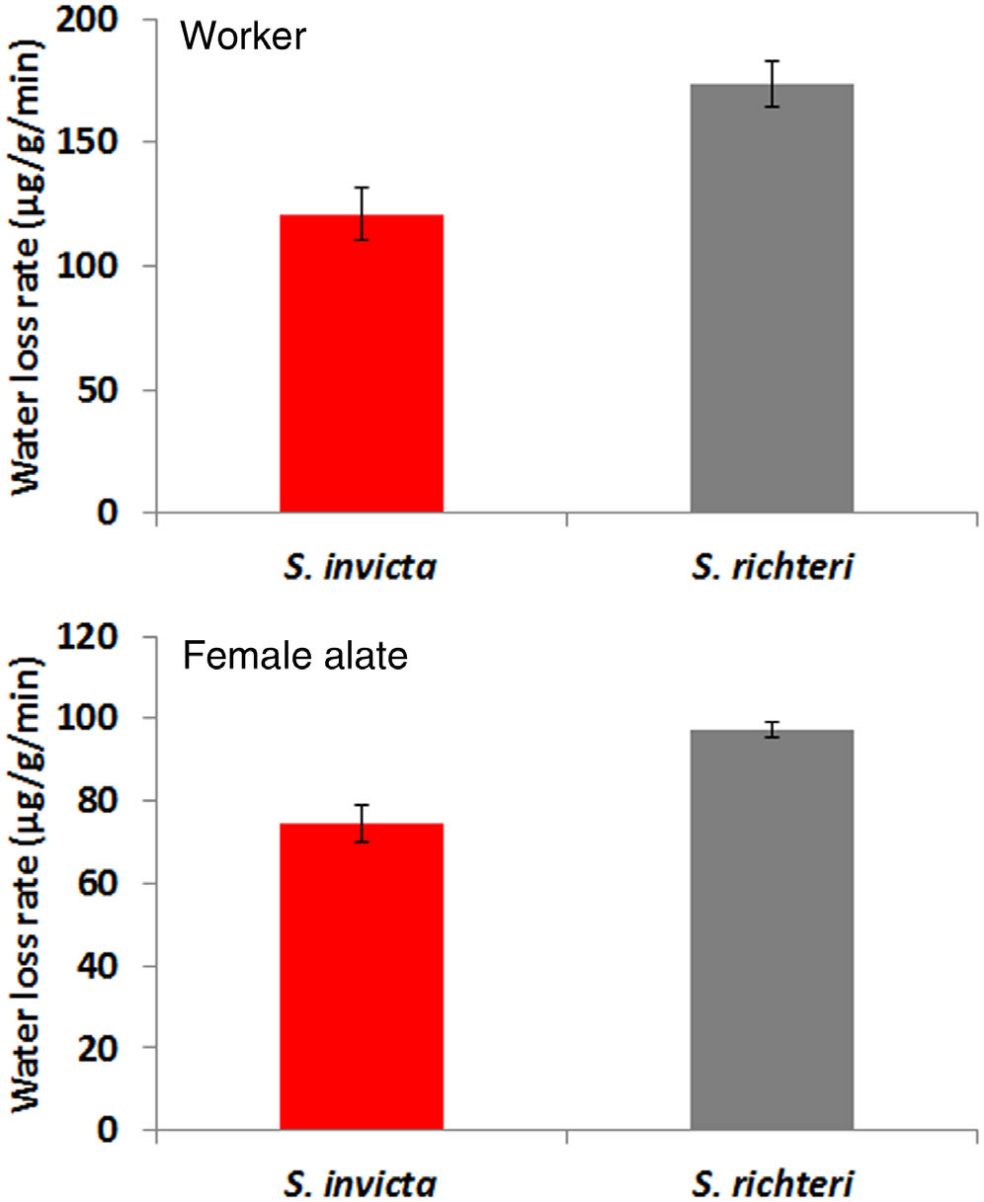
Water loss rates of workers and female alates under experimental desiccation stress (23.5% RH at 23°C). The difference between two species was significant for both workers and female alates.

For both workers and female alates, there was a significant difference in water loss rate between species (worker: *t* = 3.75; *df* = 16; *P* = 0.0017; female altes: *t* = 5.43; *df* = 30; *P*<0.0001) (Fig. 3). *S. invicta* always had a significantly lower body water loss rate than *S. richteri.*

### Total Water Content

**Figure 4:**
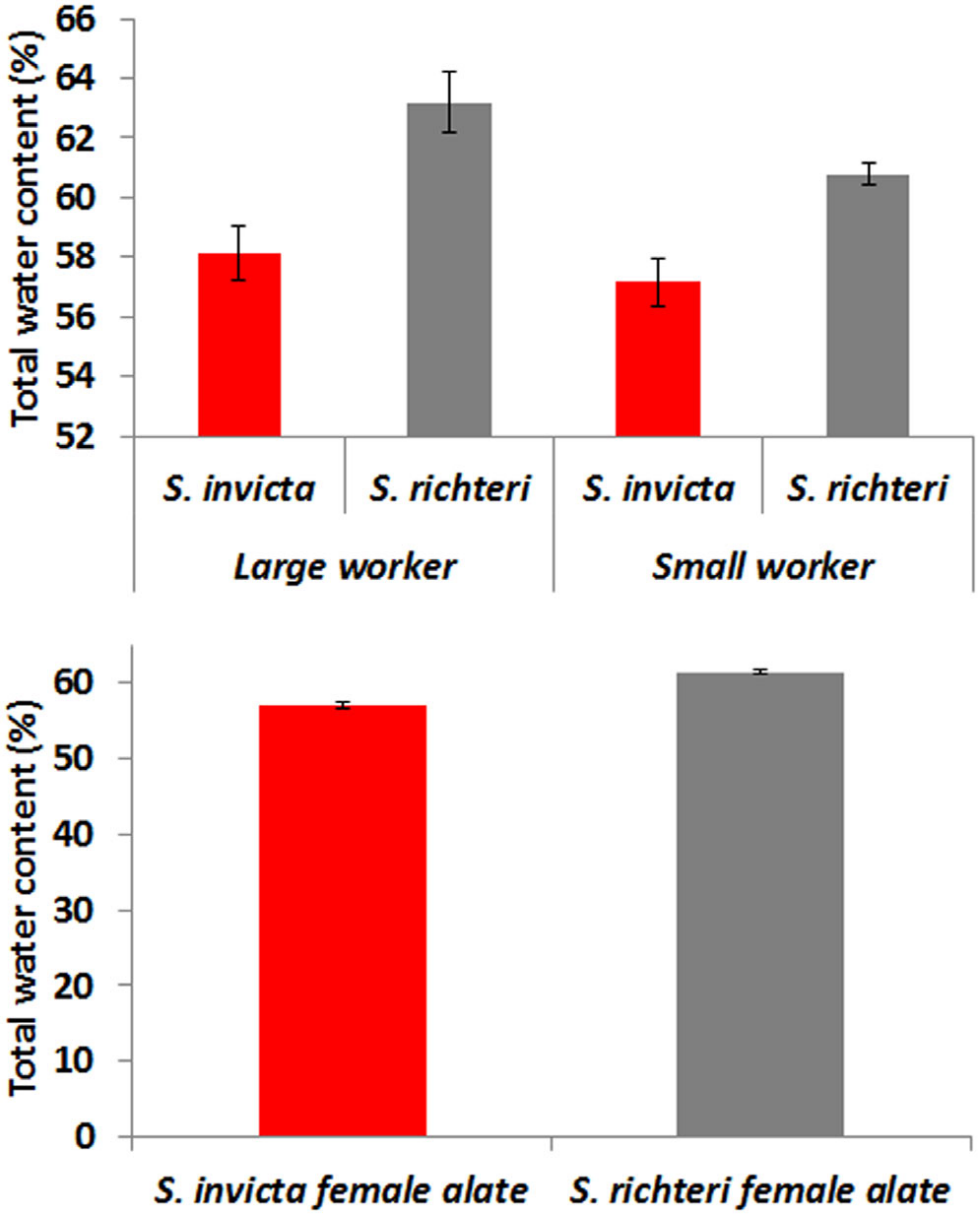
Total water content (%) of *S. invicta* and *S. richteri* workers and female alates. The difference between two species was statistically significant for both small, large workers and female alates.

*S. richteri* had significantly higher water content for both small workers (*t* = 4.05, *df = *28, *P = *0.0004) and large workers (*t* = 3.64, *df = *28, *P = *0.0011). Female alates of *S. richteri* also had significantly higher water content than *S. invicta* (*t* = 9.76, *df* = 198, *P*<0.0001) (Fig. 4).

### Critical Water Content

**Figure 5:**
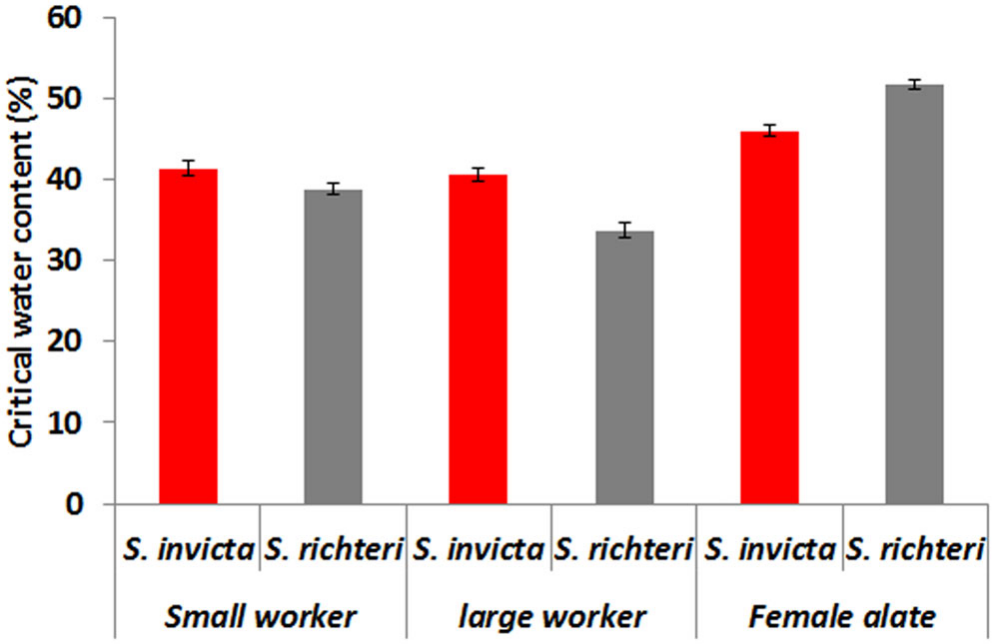
Body water content of moribund *S. invicta and S. richteri* workers under experimental desiccation stress (23.5% RH at 23°C). Small *Solenopsis richteri* had significantly lower critical water content than *S. invicta* and so did the large workers. However, female alates of *S. richteri* had significantly higer critical water content than *S. invicta*.

Small *S. richteri* workers had significantly lower critical water content than *S. invicta* (*t* = 2.60, *df* = 76, *P* = 0.011) and so did the large workers (*t* = 5.42; *df* = 87; *P*<0.0001) (Fig. 5). However, female alates of *S. richteri* had significantly higher critical water content than *S. invicta* (*z* = 5.98, *P*<0.001) (Fig. 5).

### Effect of Cuticular Lipid on Water Loss

**Figure 6:**
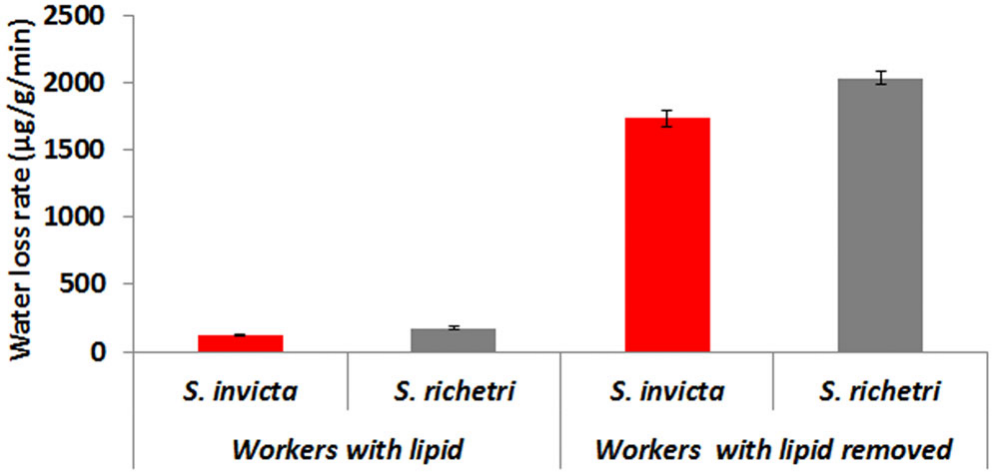
Effect of cuticular lipid on body water loss rates. Removing cuticular lipids using hexane increased the water loss rate 14.32 times for *S. invicta* workers and 11.68 times for *S. richteri* workers.

Cuticular lipids played a significant role in preventing body water loss for both species (Fig. 6). Removing cuticular lipids using hexane increased the water loss rate 14.32 times for *S. invicta* workers and 11.68 times for *S. richteri* workers. After lipids were removed from both species, *S. richteri* workers still had a significantly greater water loss rate than *S. invicta* (*t* = 3.83, *df* = 4, *P* = 0.02).

## Discussion

*S. richteri*, both workers and female alates, had significantly higher mortality than *S. invicta* under heat and desiccation stress. *S. richteri* workers had higher total water content and their lower critical water content indicated that they might have higher water loss tolerance. However, the higher water loss rate might ultimately cause *S. richteri* to survive significantly shorter than *S. invicta* under the same desiccation stress. Both *S. invicta* and *S. richteri* are mound-building ant species, and thus they are able to take advantage of a vertical gradient of temperature and humidity in the soil. However, some critical activities, such as forage, nuptial flight and colony migration, will inevitably expose them to the less stable ambient temperature and humidity. Higher heat and desiccation resistance may enable *S. invicta* to perform their critical activities in less favorable temperature and humidity conditions.

Why does *S. richteri* have significantly higher total water content than *S. invicta*? It is an interesting subject for future research. Since the same food source (sugar water and crickets) was used for maintaining colonies, such differences might not be the result of diet, as previously found in other ant species [24]. In addition to the difference in body water requirement (physiological trait), *S. richteri* might just store more fluid in the crop (behavioral trait) as for *Linepithema humile* and *Forelius mccooki* when being compared to *Myrmecocystus* spp [24]. Insects with a high fat content are generally those that also have a low water content [25]. It will be interesting to see whether *S. invicta* has a higher fat content than *S. richteri*.

A number of molecules are involved in tolerance to desiccation, and in many cases, they are also related to cold tolerance [26], [27], [28]. For example, trehalose reduced the effect of dehydration and cold damage in many terrestrial insects [29], [30], and both desiccation and low temperature stimulate the production of trehalose [26], [28], [31]. It has long been known that *S. richteri* workers were more cold tolerant than *S. invicta* [19], so it was not surprising to find that *S. richteri* workers also had significantly lower critical water content than *S. invicta*. The mechanism of water loss tolerance in *S. richteri* and its relation to cold tolerance is also an interesting venue of future research.

The cuticle is believed to be a major route of water loss in terrestrial arthropods, including ants [22], [32], [33], [34], [35]. The primary factors determining cuticular permeability are the amount and composition of the waterproofing component of the epicuticle lipids, mainly cuticular hydrocarbons [22]. This study showed that cuticular lipid did contribute to the difference in water loss rate between *S. invicta* and *S. richteri*; however, they were not the only factor accounting for differences in desiccation resistance. After epicuticular lipids were removed by hexane washing, *S. invicta* workers still had significantly lower water loss rate, suggesting that the observed difference in water loss rate was not solely based on differences in waterproofing epicuticular lipids. The composition and structure of the cuticle beneath the lipid layer might have even greater contribution.

Since regulating heat and desiccation tolerance can be very expensive, differences in traits associated with heat tolerance and water balance among species can lead to performance and ultimately fitness differences. In other words, an ant species with higher resistance to heat and desiccation stress is more likely to be successful. However, the same adaptation can be achieved through different routes. For example, some desert ants developed behavioral adaptation to avoid over-heating and desiccation without increasing the physiologic tolerance [36]. Furthermore, for a particular species, many other biological and/or ecological traits may also contribute to its invasion success. Whether the observed difference in tolerance to heat and desiccation stress is indeed associated with the variation in invasion success between *S. invicta* and *S. richteri* can only be confirmed by further extensive comparative study in biology, ecology and behavior.
